# Immunization interventions to interrupt hepatitis B virus mother-to-child transmission: a meta-analysis of randomized controlled trials

**DOI:** 10.1186/s12887-014-0307-2

**Published:** 2014-12-20

**Authors:** Hui Jin, Yueyuan Zhao, Zhaoying Tan, Xuefeng Zhang, Yaoyun Zhao, Bei Wang, Pei Liu

**Affiliations:** Department of Epidemiology and Health Statistics, Southeast University, Nanjing, China; Key Laboratory of Environmental Medicine Engineering, Ministry of Education, School of Public Health, Southeast University, Nanjing, China; Jiangsu Provincial Centre for Disease Control and Prevention, Nanjing, China

**Keywords:** Hepatitis B immunoglobulin, Hepatitis B virus, Meta-analysis, Mother-to-child transmission

## Abstract

**Background:**

This study aimed to determine the clinical efficacy of various immune interventions on mother-to-child transmission (MTCT) of hepatitis B virus (HBV).

**Methods:**

We retrieved different immune strategies on how to prevent MTCT reported in the literature from Chinese and English electronic databases from the viewpoint of intrauterine and extrauterine prevention. Relative risk (RR) and 95% confidence interval (CI) methods were used.

**Results:**

Twenty-five articles on intrauterine prevention and 16 on extrauterine prevention were included in the analysis. Intrauterine prevention could reduce infants’ HBV infection rate (RR = 0.36, 95% CI: 0.28-0.45) and increase their anti-hepatitis B surface–positive rate (RR = 2.42, 95% CI: 1.46-4.01) at birth. Compared with passive immunization, passive-active immunization could reduce infants’ HBV infection rate (RR = 0.66, 95% CI: 0.52-0.84) at birth, even at more than 12 months of age (RR = 0.54, 95% CI: 0.42-0.69). Subgroup analysis demonstrated similar results except for pregnant women who were hepatitis B surface antigen–positive. Funnel plots and Egger’s tests showed publication bias mainly in intrauterine prevention not in extrauterine one.

**Conclusions:**

The long-term protective effect of pregnant women injected with hepatitis B immunoglobulin during pregnancy should be further validated by large-scale randomized trials. Newborns of pregnant women who carried HBV should undergo a passive-active immunization strategy.

**Electronic supplementary material:**

The online version of this article (doi:10.1186/s12887-014-0307-2) contains supplementary material, which is available to authorized users.

## Background

Hepatitis B virus (HBV) infections are a global health problem [1]. Studies have shown that in neonates born to women who were hepatitis B surface antigen (HBsAg)-positive, 10–20% were infected with HBV, whereas those born to mothers who were HBsAg- and hepatitis B e antigen (HBeAg)-positive (double positive, DP), 90% were infected with HBV [2]. Mother-to-child transmission (MTCT) greatly contributes to the persistence of the high number of HBV carriers because infections occurring in neonates and in childhood result in a chronic HBV rate of 80–90% and 30–50%, respectively [3].

Since the introduction of HBV vaccines (HBVac), the use of hepatitis B immunoglobulin (HBIG) and HBVac, termed passive-active immunization, has been efficient for preventing MTCT of HBV [4-6]. In the 1980s, studies showed that in newborns of HBsAg-positive mothers, the vertical transmission rate was reduced to 23% after vaccination with HBIG [7] and to 3–7% after passive-active immunization [8]. Although a meta-analysis showed that the passive-active immunization was effective [5], Kenneth et al. [9] found that most of the studies were of low quality (e.g., lacking blinding and allocation concealment); few studies involved the effect of evaluating mothers who were HBsAg-positive and HBeAg-negative (single positive, SP).

Furthermore, 10–20% of newborns with HBsAg-positive mothers are still chronically infected with HBV, even after being vaccinated with HBIG and HBVac [10-12]. Wang et al. [13] and Zhang et al. [14] found that most immunization failures in newborns with DP mothers were due to intrauterine infection [11,15]. HBsAg does not cross the placenta, whereas HBeAg can cross the placenta and reach the fetus [15,16]. These studies suggested that intrauterine HBV infection had a close relationship with HBeAg-positive mothers, preterm birth, and HBV in the placenta [11].

Several studies in China have suggested that there are protective effects, namely lower HBV infection rates or higher anti–hepatitis B surface (HBs) levels for newborns after their mothers were injected with HBIG during pregnancy [17-19] than those in a control group included in some meta-analyses [20,21]. However, Yuan et al. [22] found that there were no significant differences in newborns between vaccination and no vaccination with HBIG during pregnancy; they also suggested that HBV intrauterine transmission was not common [23-25]. Although previous meta-analysis to support the protective effects for newborns after their mothers were injected with HBIG during pregnancy, because they ignored the randomization group, or an imbalance of HBeAg infection status in pregnancy women could have potentially biased the results. Moreover, there was serious heterogeneity in these studies because of the quality of the studies included and the infection status of the mothers [26].

Therefore, based on system review and previous meta-analysis, this study aimed to update and again evaluate the effects of different immunization interventions, including mothers injected with HBIG during pregnancy and newborns injected with HBVac and/or HBIG to interrupt the MTCT of HBV.

## Methods

### Search strategy

We searched the Medline, EMBASE, Cochrane Library, China Biological Medicine Database, Chinese National Knowledge Infrastructure, and VIP Database for Chinese Technical Periodicals databases between January 1980 and December 2013 for relevant randomized controlled trials (RCTs) written in English and Chinese peer-reviewed literature. We used the terms “HBIG” (or “hepatitis B immunoglobulin”) and “HBV” (or “hepatitis B virus”) and “intrauterine” (or “ectopic” or “pregnant” or “pregnancy” or “mother” or “children” or “infant” or “newborn”). The bibliographies of the original studies, reviews, and relevant conference abstracts were manually searched.

### Inclusion and exclusion criteria

The inclusion criteria designs or epidemiologic methods were RCTs. The subjects were HBsAg- and HBeAg-positive pregnant women or HBsAg-positive pregnant women with a clear classification of HBeAg-positive and HBeAg-negative. The experimental and control groups were comparable, and one of the following comparisons was made. (1) In the experimental group, women in the third trimester of pregnancy were injected with HBIG; newborns were injected with HBIG and HBVac. In the control group, only newborns were injected with HBIG and HBVac. (2) In the experimental group, newborns were injected with HBIG and HBVac. In the control group, only newborns were injected with HBVac. (3) In the experimental group, women in the third trimester of pregnancy were injected with HBIG; newborns were injected with HBIG and HBVac. In the control group, only newborns were injected with HBVac. Subjects were asymptomatic HBsAg carriers during the study period.

Exclusion criteria were studies without a control group and studies with a control group without randomization. Only recent or detailed studies were chosen for repeated published studies.

### Data extraction and definitions of outcome

Two researchers (HJ and YYZ) independently selected relevant studies and made a post-hoc assessment of methodological quality by means of the Cochrane library study quality evaluation tool [27]. The extracted data included the first author’s name, year of publication, study method, treatment protocol, sample size, duration of follow-up, inclusion/exclusion criteria, and relevant outcome data.

With regard to outcome, we estimated the rate of infant HBV infection (HBsAg or HBV DNA) or protection (HBsAb) at various time points (within 24 hours of birth, at 7–12 months of age, and after 12 months of age) as the primary outcome. HBV intrauterine infection was defined as HBsAg and/or HBV DNA positivity in neonatal peripheral or umbilical blood within 24 hours of birth and before administration of active or passive immune prophylaxis. HBsAg-positive infections were classified as events (HBsAg-positive at any time >1 month of age) or as chronic (HBsAg-positive for 6 months).

### Quality assessment

The quality of the studies was evaluated using the Cochrane Handbook for Systematic Reviews of Interventions (Additional file 1: Table S1), version 5.1.0, recommended standard: random sequence generation, allocation concealment, blinding, incomplete outcome data, selective reporting, and other biases. The risk of bias was regarded high in the presence of high bias in any domain, low if all key domains (except random sequence generation and allocation concealment) were of low bias, and unclear in all other cases. Two authors (HJ and ZT) independently assessed the risk of bias; when necessary, consensus was determined through help of a third author (PL).

### Statistical analysis

Statistical analysis was performed according to the intention-to-treat principle. The estimated pooled relative risk (relative ratio, RR) and 95% confidence interval (95% CI) were determined by the Mantel–Haenszel fixed-effects model, or the inverse variance random-effects model. The heterogeneity test was used with the chi-squared test and *I*^*2*^. An *I*^*2*^ index of 25%, 50%, and 75% indicated a low, moderate, and high degree of heterogeneity, respectively. P < 0.10 in the chi-squared test showed the existence of heterogeneity between studies.

Subgroup analysis included mothers with HBeAg status, the length of follow-up, and the quality of the included study. The Begg’s [28] and Egger’s [29] methods were used to check for publication bias. For all tests, P ≤ 0.05 or 95% CIs not including “1” indicated statistical significance. The statistical analysis software used was RevMan 5.1.0 (Copenhagen: Nordic Cochrane Centre, The Cochrane Collaboration, 2011).

## Results

### Search results

**Figure 1:**
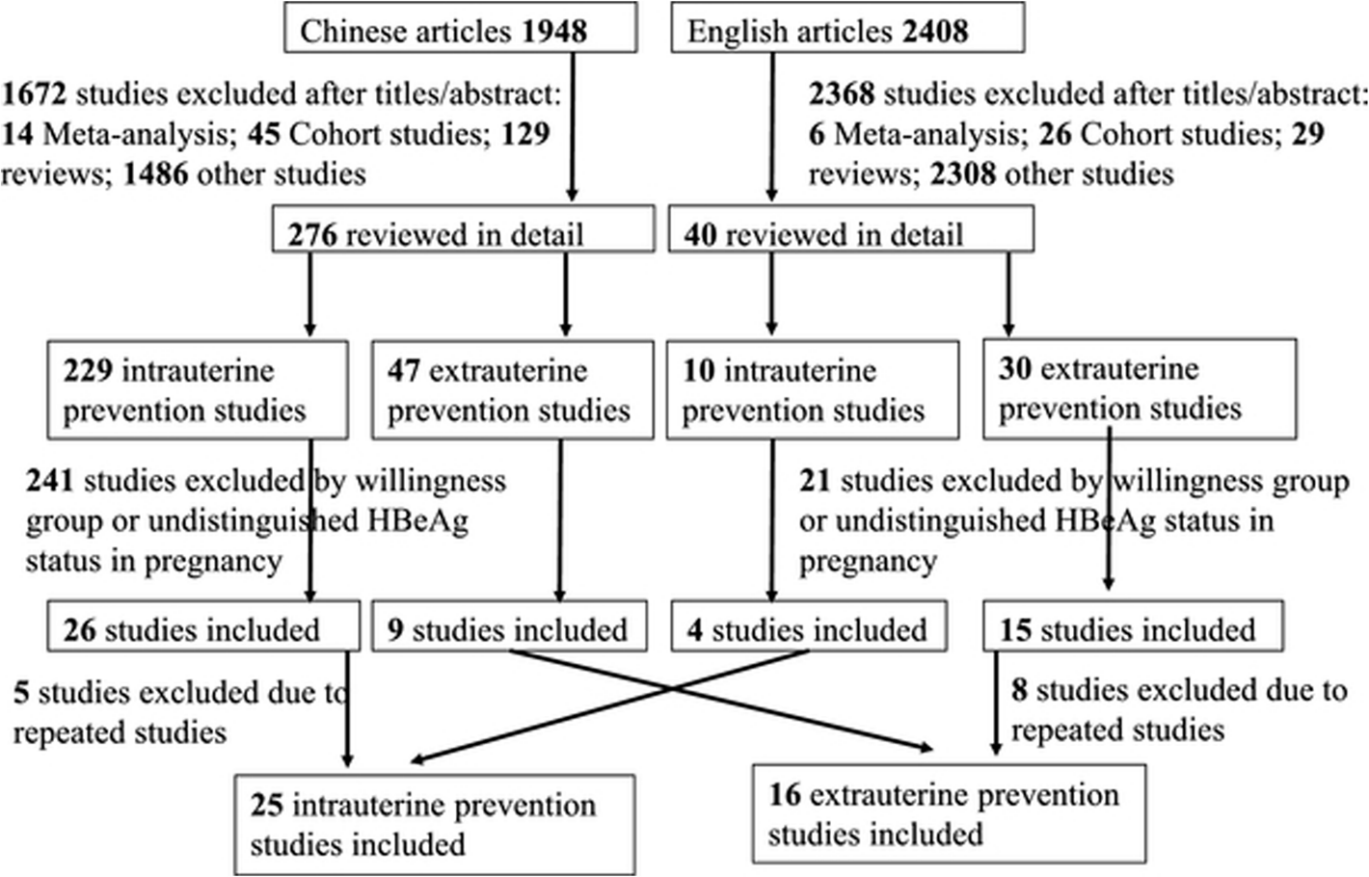
**Flow chart of included studies.**

Figure 1 is a flow chart of the included studies. The number of RCT studies on intrauterine and extrauterine prevention was 30 [22,30-58] and 24 [8,12,59-80], respectively. Among studies on intrauterine prevention, five were excluded because of duplicate publication and the remaining 25 (eight on mothers who were DP, 17 on those who were HBsAg- and/or HBeAg-positive), which were conducted in the mainland of China, were included. Among the studies on extrauterine prevention, eight studies were excluded because of duplicate publication, and the remaining 16 (13 on mothers who were HBsAg- and HBeAg-positive, three on those who were HBsAg- and/or HBeAg-positive) were included. The characteristics of included studies are shown in Tables 1 and 2.

**Table 1:** **Characteristics of intrauterine and extrauterine prevention for newborns born to HBsAg- and/or HBeAg-positive women**

**Reference**	**Mothers’ age (years)**	**E** ^**1)**^	**Immune prophylaxis**		**Sample size**	**Newborn**		**7-12 month infant**		**>12 month child**	
			**Mother (schedule/pregnancy month)**	**Child (schedule/infant month)**		**HBsAg+**	**HBsAb+**	**HBsAg+**	**HBsAb+**	**HBsAg+**	**HBsAb+**
Ji 2003 [30]	21-31	1	T: HBIG 200 IU (7,8,9)	NR	T:29	T:3	T:10	NR	NR	NR	NR
			C: none		C:31	C:5	C:3				
Xu 2006 [31]	NR	1	T: HBIG 200 IU (7,8,9)	NR	T:30	T:7	NR	NR	NR	NR	NR
Repeated [32]			C: none		C:30	C:20					
Yuan 2006 [22]	20-33	1	T: HBIG 400 IU (7,8,9)	T : HBIG 200 IU(0) + RV 5 ug(0,1,6)	T:118	T:27	T:0	T:13	T:101	NR	NR
			C: Diluent	C: HBIG 200 IU(0) + RV 5 ug(0,1,6)	C:113	C:32	C:0	C:17	C:112		
Chen 2007 [33]	NR	1	T1: HBIG 200 IU (7,8,9)	T1: HBIG 200 IU(0,0.5) + RV 5 ug(0,1,6)	T1:45	T1: 1	T1: 14	T1: 1	T1:33	NR	NR
			T2: None	T2: HBIG 200 IU(0,0.5) + RV 5 ug(0,1,6)	T2:44	T2: No	T2: No	T2: 3	T2: 35		
			C:None	C: RV 5ug(0,1,6)	C:49	C:13	C: 4	C: 13	C: 32		
Sun 2007 [34]	NR	1	T1: HBIG 200 IU (7,8,9)	T1: HBIG 200 IU(0,0.5) + V 5 ug(0,1,6)	T1:77	T1: 2	NR	T1:1	T1: 73	T1:0	T1: 54
			T2:None	T2: HBIG 200 IU(0,0.5) + V 5 ug(0,1,6)	T2:76	T2: 10		T2: 4	T2: 70	T2:1	T2: 50
			C: None	C : V 5ug(0,1,6)	C:70	C: 9		C: 8	C: 58	C: 4	C: 30
Wang 2007 [35]	NR	1	T: HBIG 200 IU (4–9)	T: HBIG 200 IU(0,0.5) + V 10 ug(1,2,7)	T:32	T: 2	NR	T: 2	NR	NR	NR
			C: none	C: HBIG 200 IU(0,0.5) + V 10 ug(1,2,7)	C:31	C: 11		C: 12			
Yan 2009 [36]	22-35	1	T: HBIG 400 IU (7,8,9)	T: HBIG 200 IU(0,0.5) + RV 10 ug(0,1,6)	T:106	T:10	T:37	T: 9	T: 82	T:8	T: 93
			C: none	C: RV 10ug(0,1,6)	C:98	C:23	C: 9	C: 21	C: 46	C:20	C: 69
Cui 2011 [37]	NR	1	T: HBIG 200 IU, 3 time	T: HBIG 100 IU, 2time + RV 5 ug, 0, 1,6	T:106	NR	NR	NR	NR	T:5	T:96
			C: none	C: RV 5 ug, 0, 1,6	C:82					C:16	C:60
Zhu 1997 [38]	NR	1	T: HBIG 200 IU (7,8,9)	NR	T:37	T: 6	NR	NR	NR	NR	NR
					C:32	C: 12					
Repeated [39]		2	C: none		T:68	T: 0	NR	NR	NR	NR	NR
					C:70	C: 3					
Jia 2001 [40]	NR	1	T: HBIG 200 IU (7,8,9)	NR	T:15	T: 1	NR	NR	NR	NR	NR
					C:16	C: 7					
		2	C: none		T:25	T: 0	NR	NR	NR	NR	NR
					C:30	C: 3					
Chi 2002 [41]	NR	1	T: HBIG 200 IU (7,8,9)	NR	T:27	T:4	NR	NR	NR	NR	NR
					C:29	C:10					
		2	C: none		T:42	T: 0	NR	NR	NR	NR	NR
					C:43	C:2					
Chen2003 [42]	NR	1	T: HBIG 200 IU (7,8,9)	NR	T:18	T:2	NR	NR	NR	NR	NR
					C:15	C:6					
		2	C: none		T:26	T: 1	NR	NR	NR	NR	NR
					C:20	C: 2					
Han 2003 [43]	NR	1	T: HBIG 200 IU (7,8,9)	T: HBIG 200 IU(0,0.5) + V 5 ug(1,2,7)	T:83	T:21	NR	T: 5	NR	NR	NR
					C:52	C:23		C: 7			
Repeated [44]		2	C: None	C : HBIG 200 IU(0,0.5) + V 5 ug(1,2,7)	T:43	T: 3	NR	T: 0	NR	NR	NR
					C:38	C: 9		C: 5			
Xing 2003 [45]	22-38	1	T: HBIG 200 IU (7,8,9)	NR	T:16	T: 2	NR	NR	NR	NR	NR
					C:15	C: 6					
Repeated [46]		2	C: None		T:30	T: 0	NR	NR	NR	NR	NR
					C:25	C: 3					
Zhu 2003 [47]	NR	1	T: HBIG 200-400 IU (7,8,9)	T: HBIG 100 IU(0,0.5) + RV 5 ug(1,2,7) or PDV 30 ug(1,2,7)	T:169	T:21	NR	NR	NR	NR	NR
					C:189	C:49					
Repeated [48]		2	C: none	C: HBIG 100 IU(0,0.5) + RV 5ug(1,2,7) or PDV 30 ug(1,2,7)	T:318	T:7	NR	NR	NR	NR	NR
					C:304	C:22					
Chen 2006 [49]	NR	1	T: HBIG 200 IU (7,8,9)	NR	T:16	T: 4	NR	NR	NR	NR	NR
					C:14	C: 9					
		2	C: none		T:34	T: 1	NR	NR	NR	NR	NR
					C:36	C: 5					
Yang 2006 [50]	NR	1	T: HBIG 200 IU (4–9)	NR	T:117	T: 12	T:7	NR	NR	NR	NR
			C: None		C:90	C: 48	C: 0				
		2	T: HBIG 200 IU (7,8,9)		T:46	T: 2	T:10	NR	NR	NR	NR
			C: None		C:32	C:14	C: 0				
Yu 2006 [51]	NR	1	T1: HBIG 200-400 IU (7–10)	NR	T1:8	T1:3	NR	NR	NR	NR	NR
					T2:7	T2:5					
					C:8	C: 8					
		2	T2: HBIG 200 IU (7,8,9)		T1:18	T1:0	NR	NR	NR	NR	NR
					T2:22	T2:0					
					C:20	C:2					
			C: Diluent								
Ji 2007 [52]	NR	1	T: HBIG 200 IU (7,8,9)	T: HBIG 200 IU(0) + RV 5 ug(0,1,6)	T:30	T:2	NR	T: 1	NR	NR	NR
					C:26	C:10		C: 6			
		2	C: None	C: HBIG 200 IU(0,0.5) + RV 5 ug(0,1,6)	T:83	T:3	NR	T: 1	NR	NR	NR
					C:84	C:5		C: 3			
Liu 2007 [53]	NR	1	T: HBIG 200 IU (7,8,9)	T: HBIG 200 IU(0,0.5) + RV 10 ug(0,1,6)	T:12	T: 1	T: 4	T: 0	T:10	NR	NR
					C:9	C: 2	C: 1	C: 2	C: 4		
		2	C: None	C: HBIG 200 IU(0,0.5) + RV 10 ug(0,1,6)	T:31	T: 1	T: 12	T: 0	T:24	NR	NR
					C:34	C: 1	C: 12	C: 1	C:25		
Wang 2008 [54]	20-33	1	T:HBIG 200 IU(5–9)	T: HBIG 200 IU(0,0.5) + V	T:79	T:8	NR	T: 7	NR	NR	NR
					C:60	C: 19		C: 14			
		2	C: None	C:V	T:80	T: 2	NR	T: 0	NR	NR	NR
					C:60	C:8		C: 5			
Zhao 2008 [55]	20-34	1	T: HBIG 200 IU (7,8,9)	NR	T:37	T: 6	NR	NR	NR	NR	NR
					C:32	C:12					
		2	C: none		T:66	T: 0	NR	NR	NR	NR	NR
					C:69	C:3					
Liu 2009 [58]	NR	1	T: HBIG 200 IU (7,8,9)	NR	NR	NR	NR	NR	NR	NR	NR
		2	C: none		T:100	T: 1	NR	NR	NR	NR	NR
					C:120	C:4					
Yuan 2009 [57]	20-40	1	T1: HBIG 200 IU (7,8,9)	T1: HBIG 200 IU(0,0.5) + RV 5 ug(0,1,6)	T1:4	NR	NR	T1: 0	T1:13	NR	NR
					T2:9			T2: 3	T2: 7		
			T2: None	T2: HBIG 200 IU(0,0.5) + RV 5 ug(0,1,6)	C:13			C: 5	C: 10		
		2	C: None	C: RV 5 ug(0,1,6)	T1:23	NR	NR	T1:1	T1:23	NR	NR
					T2:13			T2:2	T2:12		
					C:13			C: 1	C: 7		
Li 2013 [58]	24-35	1	T: HBIG 200 IU (7,8,9)	T: HBIG 100 IU, 6 h + RV 10 ug, 0, 1,6	T:38	T:2	T:34	T:0	T:36	NR	NR
					C:34	C:12	C:15	C:11	C:15		
		2	C: none	C: HBIG 100 IU, 6 h + RV 10 ug, 0, 1,6	T:14	T:0	T:13	T:0	T:14	NR	NR
					C:28	C:3	C:20	C:1	C:25		

^1)^E = HBeAg, 1 refers to pregnancy with HBeAg and HBsAg positivity; ^2^refers to pregnancy with HBsAg positivity and HBeAg negativity.

T, experimental group; C, control group.

V, vaccine; PDV, plasma-derived vaccine; RV, recombinant vaccine; HBIG, hepatitis B immunoglobulin; NR, not reported.

**Table 2:** **Characteristics of extrauterine prevention alone for newborns born to HBsAg- and/or HBeAg-positive women**

**Reference**	**E** ^**1)**^	**Infant’s Immune prophylaxis** ^**2)**^ **(schedule/month )**	**Sample size (n)**	**Newborn**		**7-12month infant**		**>12 month child**	
				**HBsAg-pos**	**HBsAb-pos**	**HBsAg-pos**	**HBsAb-pos**	**HBsAg-pos**	**HBsAb-pos**
Lo 1985 [59-61]	1	T: HBIG50 IU(0) + PDV5ug	T:36	NR	NR	T:4	T:32	NR	NR
		(0.5,1.5,2.5); C: PDV5ug (0.5,1.5,2.5)	C:38			C: 9	C:30		
Sha 1985 [62]	1	T: HBIG 0.5 ml(0) + PDV 20ug (0,1,2,12);	T:19	T:13	T:18	T:4	T :10	NR	NR
		C: PDV 20 ug(0,1,2,12)	C:10	C:7	C:0	C:1	C:5		
Wu 1986 [63]	1	T : HBIG 1 ml(0) + PDV 20ug (1,2,3)	T:13;	T:1;	NR	NR	NR	NR	NR
		C : PDV 20 ug(1,2,3)	C:6	C:0					
Farmer 1987 [64]	1	T: HBIG 0.25 ml (25 IU/kg)(0,1.5),	T:21	NR	NR	T:3	T:17	NR	NR
		PDV5 ug(0,1.5, 6) C: PDV5 ug (0,1.5,6)	C:18			C:4	C:13		
Theppisai 1987 [65]	1	T: HBIG 200 IU(0) + PDV 10 ug (0,1,6)	T:27	NR	NR	T:2	NR	NR	NR
		C: PDV 10 ug(0,1,6)	C:18			C:2			
Ip1 989 [8,66,67]	1	T: PDV3 ug(0,1,2,6) + HBIG(0)	T:64	NR	NR	T:8	NR	T:9	T:47
		C: PDV3 ug(0,1,2,6)	C:64			C:15		C:15	C:52
Assateerawatt 1993 [68]	1	T:HBIG100IU(0) + RV20 ug (0,1,2,12)	T:30	NR	NR	T:1	T:25	T:1	T:24
		C: RV20 ug(0,1,2,12)	C:30			C:2	C:22	C:3	C:21
Li 1994 [69]	1	T : HBIG 200 IU(0) + PDV (0,1,6)	T1:20; C1:22	T1:7; C1: 7;	T1:18;C1:3	T1:1; C1:3	T1:16; C1:19	NR	NR
		C : PDV (1,2,3).	T2:20; C2:21	T2:7; C2: 8;	T2:9; C2:2	T2:7; C2:7	T2:17; C2:11		
		PDV including 10 ug, 20 ug and 30 ug	T3:22; C3:21	T3:7; C3: 7	T3:9; C3:2	T3:1; C3:2	T3:20; C3:20		
Zhao 1994 [70]	1	T : HBIG 60 IU(0) + V 10 ug (0,1,6)	T:40	T:2	T:35	T:2	T:36	NR	NR
		C : V 10 ug(0,1,6)	C:26	C:5	C:9	C:7	C:15		
Kang 1995 [71]	1	T: HBIG 200 IU(0,1) + RV1 10 ug (0,1,6)	T:44	NR	NR	NR	NR	T: 0	T: 43
		C: RV1 20 ug(0,1,6)	C:41					C: 5	C: 35
Poovorawan 1997 [72-74]	1	T: HBIG 100 IU(0) + RV 10 ug (0,1,6,60)	T:64	T:1	NR	T: 0	T: 58	T:1	T:39
		C: RV 10 ug(0,1,6,60)	C:63	C:3		C:3	C: 54	C:3	C:35
Lin 2000 [75]	1	T : HBIG 50 IU(0) + RV 10 ug (0,1,6)	T:31	T:4	T: 26	NR	NR	NR	NR
		C : RV 10 ug(0,1,6)	C:39	C:2	C: 36				
Meng 2001 [76]	1	T : HBIG 50 IU(0) + RV 10 ug (0,1,6)	T:50	NR	NR	NR	NR	T:4	T:45
		C : RV 10 ug(0,1,6)	C:52					C:7	C:43
Wang 2000-2001 [77,78]	1	T: HBIG 100 IU(0,1) + RV 20 ug (1,2,7)	T:104; C:241	T:20; C :76	NR	T:21; C:81	NR	T:26; C:96	NR
	2	C: PDV 20 ug(0,1,6)	T:157; C:122	T:19; C:25	NR	T:22; C:26	NR	T:28;C:35	NR
Sehgal 1992 [79,80]	1^**3)**^	T: HBIG0.5 ml(0) + PDV10 ug(0,1,2)	T:7; C:7	NR	NR	T:1; C:1	T:5;C:4	NR	NR
	2	C: PDV10 ug(0,1,2)	T:17:C:14	NR	NR	T:1; C:1	T:13;C:13	NR	NR
Xu 1995 [12]	1	T: HBIG 250 IU(0) + PDV 20 ug(0,1,6)	T:11; C:31	NR	NR	T:1; C:10	NR	T:1; C:10	NR
	2	C: PDV 20 ug(0,1,6)	T:17; C:29	NR	NR	T:0; C:2	NR	T:1; C:2	NR

^1)^E = HBeAg, 1 refers to pregnancy with HBeAg and HBsAg positivity; ^2^refers to pregnancy with HBsAg positivity and HBeAg negativity. ^2)^ Vaccination schedule is filled in () by the unit of month.

T, experimental group; C, control group.

V, vaccine; PDV, plasma-derived vaccine; RV, recombinant vaccine; HBIG, hepatitis B immunoglobulin; NR, not reported. ^3)^Six newborns infected with HBV at birth were excluded owing to the absence of intervention.

### Quality assessment

**Figure 2:**
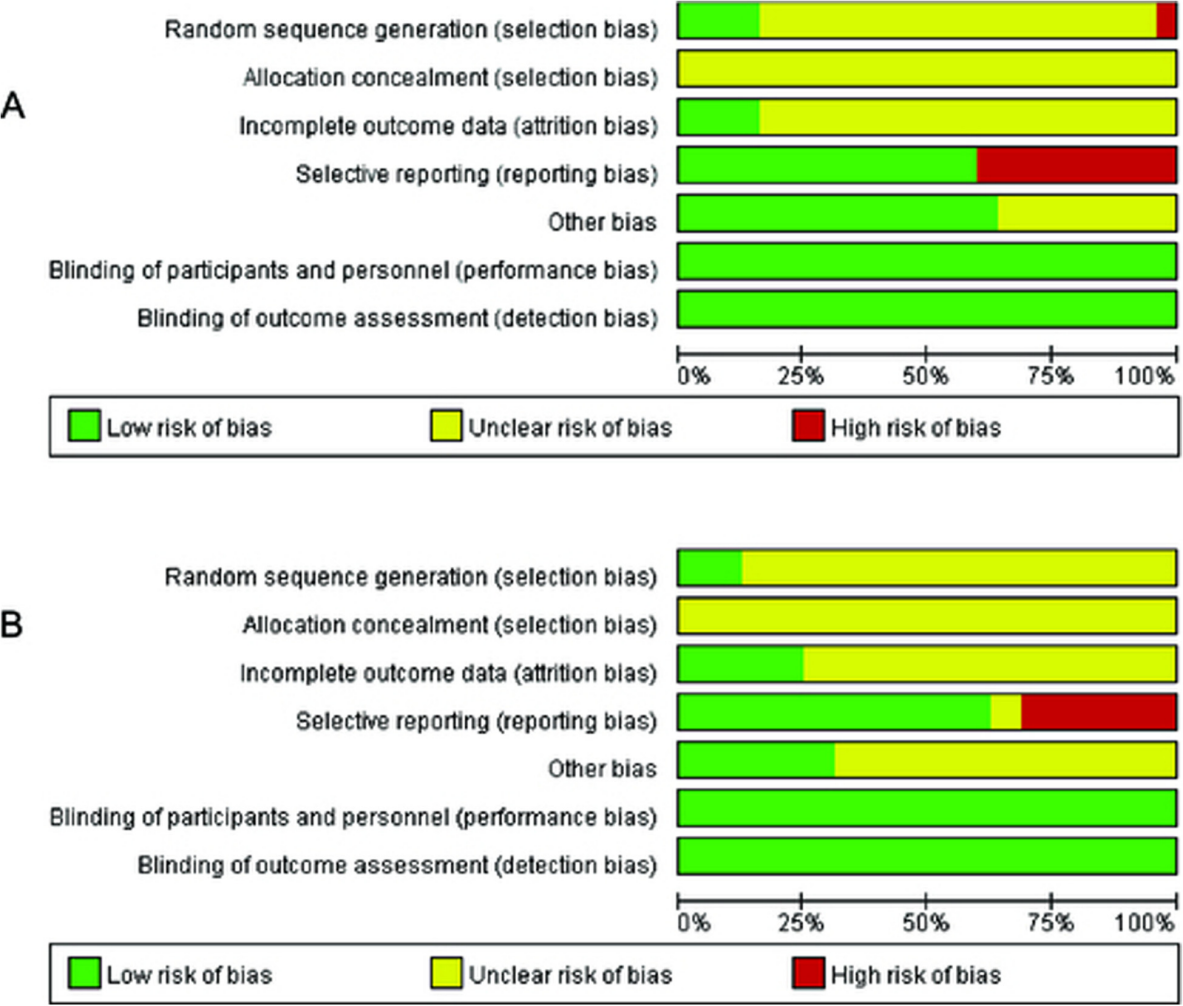
**Risk of bias graph of included studies about intrauterine and extrauterine prevention. A**. Intrauterine prevention.** B**. Extrauterine prevention.

In intrauterine prevention (Figure 2A and Additional file 2: Figure S1A), four studies indicated that a random table was applied [22,32,35,54], whereas the remainder did not report the details of random-sequence generation. Allocation concealment was an undefined risk in the included studies because it was not reported. Four studies had a low attrition bias [22,32,36,51]; others were unclear. Performance and detection biases were low. Ten studies had high risk of reporting bias because of selective reporting.

In extrauterine prevention (Figure 2B and Additional file 2: Figure S1B), two studies indicated that a random table was applied [64,68], whereas the remainder did not report the details of random-sequence generation. All allocation concealment was unclear. Four studies had a low attrition bias [12,61,67,80]; others were unclear. Performance and detection biases were low.

### Meta-analysis results

#### Intrauterine and extrauterine prevention studies

**Figure 3:**
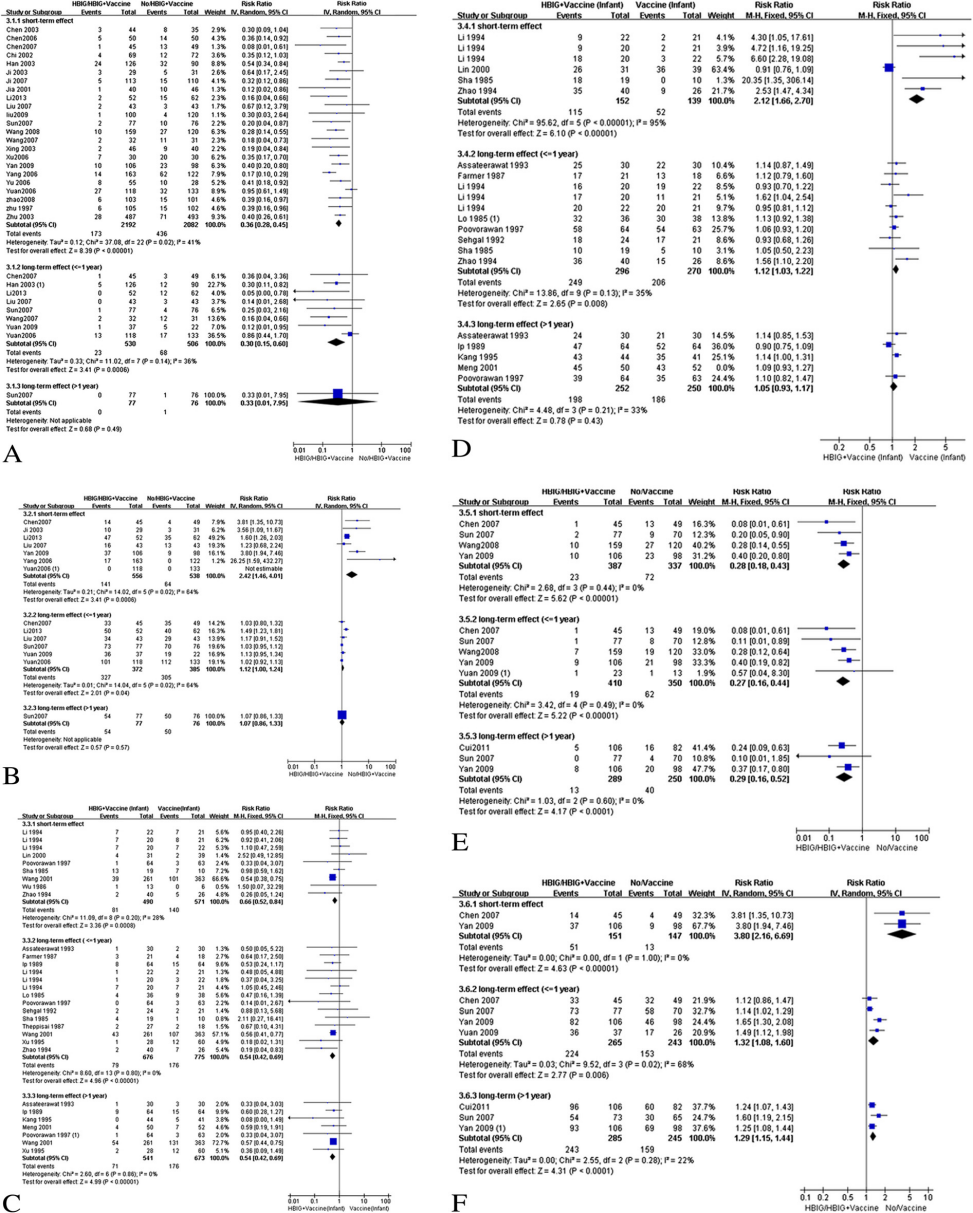
**Forest plot of HBV infection rates or the anti-HBs positive rate. (A)** Forest plot of HBV infection rates of infants born to mothers with HBsAg and/or HBeAg positive for intrauterine and extrauterine prevention (mother: HBIG/infants: HBIG + vaccine vs mother: none/infants: HBUG + vaccine). **(B)** Forest plot of the anti-HBs positive rate of infants born to mothers with HBsAg and/or HBeAg positive for intrauterine and extrauterine prevention (mother: HBIG/infants: HBIG + vaccine vs mother: none/infants: HBUG + vaccine). **(C)** Forest plots of the HBV infection rate of infants born to mothers with HBsAg and/or HBeAg positive in extrauterine prevention. **(D)** Forest plot of the anti-HBs positive rate of infants born to mothers with HBsAg and/or HBeAg positive in extrauterine prevention. **(E)** Forest plot of the HBV infection positive rate of infants born to mothers with HBsAg and/or HBeAg positive for intrauterine and extrauterine prevention (mother: HBIG/infants: HBIG + vaccine vs mother: none/infants: vaccine). **(F)** Forest plot of the anti-HBs rate of infants born to mothers with HBsAg and/or HBeAg positive for intrauterine and extrauterine prevention (mother: HBIG/infants: HBIG + vaccine vs mother: none/infants: vaccine).

Table 3 and Figure 3A show the comparison of immunization effects on newborns of HBV-infected women injected with HBIG and those without HBIG during pregnancy; they also show all of the newborns were injected with HBIG and HBVac. A total of 2192 newborns in the experimental group and 2082 in the control group at birth were included in 23 RCTs (Table 3 and Figure 3A). The meta-RR (95% CI) comparing these two groups for newborn HBsAg infection rate was 0.36 (0.28, 0.45), and a medium level of heterogeneity was observed (*I*^*2*^ = 41%). There were 530 infants in the experimental group and 506 in the control group who had data on their serum HBsAg status at 7–12 months of age that were included in eight RCTs, with a meta-RR (95% CI) of 0.34 (0.22, 0.53) (*I*^*2*^ = 36%). However, only one RCT included those at more than 12 months of age (3 years), with a meta-RR (95% CI) of 0.33 (0.01, 7.59). In subgroup analysis, there were similar protective effects as in these results, whether for maternal HBeAg status or for low risk and unclear bias (Table 3).

**Table 3:** **Comparison of immunization effects on intrauterine and extrauterine prevention in newborns of HBsAg- and/or HBeAg-positive women** ^**1)**^

**Pregnancy infection status**	**Newborn infection status**	**Detective time**	**Number of included studies**	**Sample size**	**Meta-RR (95% CI)**
Total (HBsAg+ and/or HBeAg+)	HBsAg+	At birth	23	4274	**0.36(0.28, 0.45)** ^**2)**^
		7-12 month	8	1036	**0.34(0.22, 0.53)** ^**2)**^
		>12 month	1	153	0.33(0.01, 7.95)^2)^
	HBsAb+	At birth	7	1094	**2.42(1.46, 4.01)** ^**2)**^
		7-12 month	6	757	1.12(1.00, 1.24)^2)^
		>12 month	1	153	1.07(0.86, 1.33)^2)^
Subgroup (HBsAg+ and HBeAg+)	HBsAg+	At birth	21	2159	**0.40(0.32, 0.51)** ^**2)**^
		7-12 month	8	812	**0.37(0.23, 0.57)** ^**3)**^
		>12 month	1	153	0.33(0.01, 7.95)^2)^
	HBsAb+	At birth	7	909	**3.05(2.19, 4.25)** ^**2)**^
		7-12 month	6	614	1.15(0.99, 1.34)^2)^
		>12 month	1	153	1.07(0.86, 1.33)^2)^
Subgroup (HBsAg+ and HBeAg-)	HBsAg+	At birth	15	1892	**0.22(0.14, 0.35)** ^**3)**^
		7-12 month	4	224	**0.22(0.06, 0.84)** ^**3)**^
	HBsAb+	At birth	3	185	1.31(0.82, 2.10)^3)^
		7-12 month	3	148	1.09(0.97, 1.22)^3)^
Subgroup (low+unclear bias)	HBsAg+	At birth	12	1945	**0.35(0.22,0.54)** ^**2)**^
		7-12 month	5	643	**0.52(0.30,0.91)** ^**3)**^
		>12 month	1	153	0.33(0.01,7.95)^3)^
	HBsAb+	At birth	6	980	**3.02(1.48,6.15)** ^**2)**^
		7-12 month	5	643	1.04(0.98,1.10)^3)^
		>12 month	1	153	1.07(0.86,1.33)^3)^
Subgroup (high risk bias)	HBsAg+	At birth	11	2329	**0.37(0.29,0.47)** ^**3)**^
		7-12 month	3	393	**0.18(0.08,0.39)** ^**3)**^
	HBsAb+	At birth	1	114	**1.60(1.26,2.03)** ^**3)**^
		7-12 month	1	114	**1.49(1.23,1.81)** ^**2)**^

^1)^(Mother: HBIG/Infants: HBIG + vaccine) vs (Mother: none/Infants: HBIG + vaccine); ^2)^Random effects model, inverse variance method; ^3)^Fixed effects model, Mantel-Haenszel method; values in boldface indicate statistical significance (*P* < 0.05).

Meta-analysis showed that newborns in the experimental group had a higher amount of protective antibodies at birth, but not at the other time points (95% CI including “1”), compared with the control group (Figure 3B). There were 556 newborns in the experimental group and 538 in the control group at birth, included in seven RCTs (Table 3 and Figure 3B). The meta-RR (95% CI) comparing these two groups for newborn anti-HBs–positive rate at birth was 2.42 (1.46, 4.01), and a medium level of heterogeneity was observed (*I*^*2*^ = 64%). A total of 372 infants in the experimental group and 380 infants in the control group had data on their serum anti–HBs-positive status at 7–12 months of age were included in six RCTs, with a meta-RR (95% CI) of 1.10 (0.99, 1.23) (*I*^*2*^ = 68%). However, only one RCT included those who were older than 12 months of age (3 years), with a meta-RR (95% CI) of 1.07 (0.86, 1.33). In a subgroup analysis, there were similar protective effects as these results between the experimental and control groups, whether for maternal HBeAg status or for low risk and unclear bias (Table 3).

**Figure 4:**
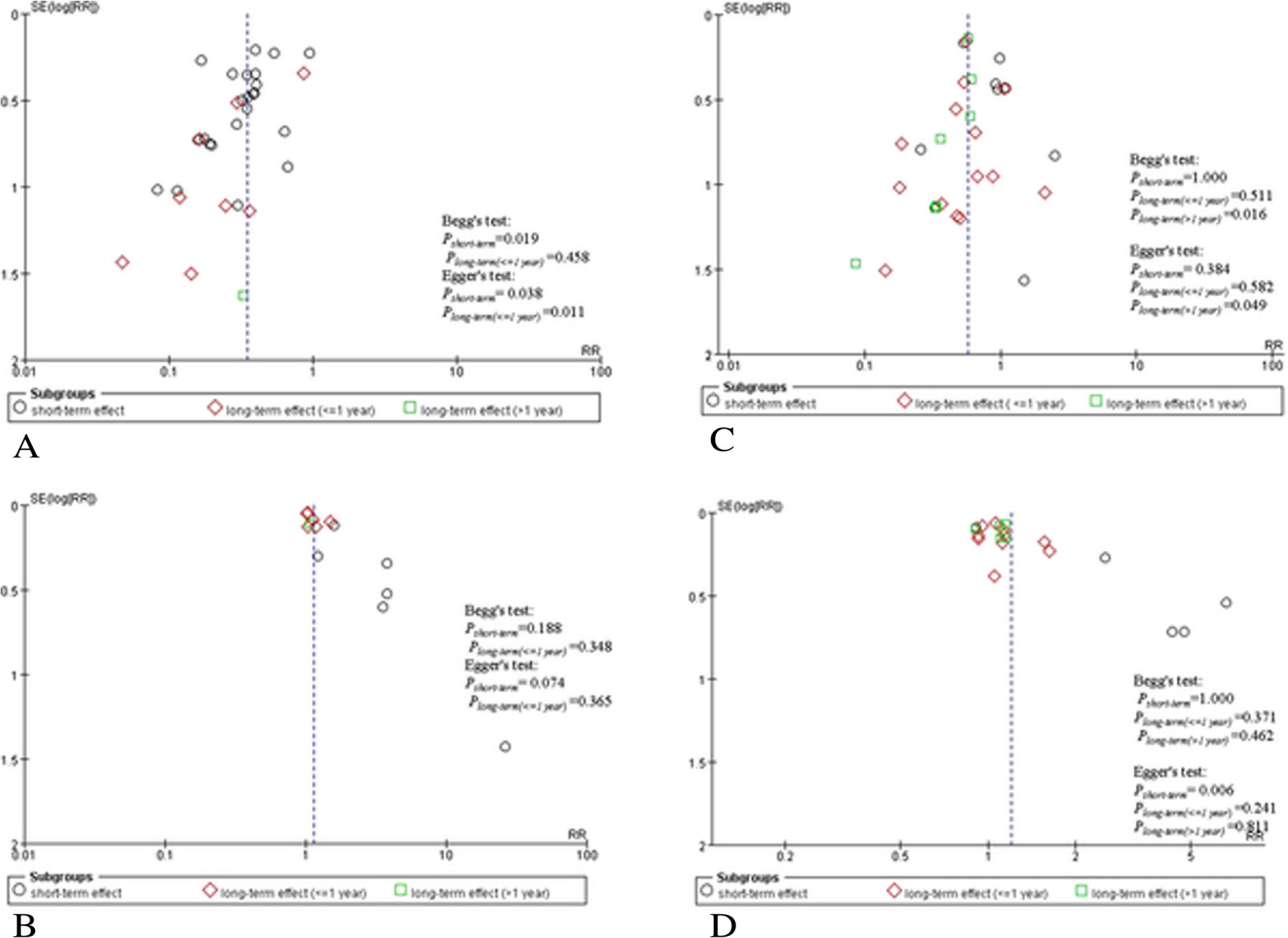
**Funnel plot of HBV infection rate or anti-HBs positive rate. (A)** Funnel plot of HBV infection rate of infants born to mothers with HBsAg and/or HBeAg positive for intrauterine and extrauterine prevention. **(B)** Funnel plot of the anti-HBs–positive rate of infants born to mothers with HBsAg and/or HBeAg positive for intrauterine and extrauterine prevention. **(C)** Funnel plot of HBV infection rate of infants born to mothers with HBsAg and/or HBeAg positive for intrauterine and/or extrauterine prevention. **(D)** Funnel plot of the anti-HBs–positive rate of infants born to mothers with HBsAg and/or HBeAg positive for intrauterine and/or extrauterine prevention.

All of the Begg’s tests, Egger’s tests, and funnel plots revealed the existence of publication bias when comparing two groups for newborn HBsAg infection rate at birth and at 7–12 months of age (Figure 4A, B). Furthermore, the funnel plot was more skewed for the groups with HBV infection rate than for those with an anti–HBs-positive rate.

### Extrauterine prevention studies

Table 4 and Figure 3C show the effects of immunization between newborns injected with HBIG and HBVac and those who were vaccinated with only HBVac and whose mothers did receive HBIG injections during pregnancy. There were 490 newborns in the experimental group and 571 in the control group at birth in seven RCTs. The meta-RR (95% CI) comparing these two groups for newborn HBsAg infection rate was 0.66 (0.52, 0.84); a low level of heterogeneity was observed (*I*^*2*^ = 28%). There were 677 infants in the experimental group and 776 in the control group with serum HBsAg status at 7–12 months of age that were included in 12 RCTs, with a meta-RR (95% CI) of 0.54 (0.42, 0.69) (*I*^*2*^ = 0%). Seven RCTs included data at more than 12 months of age, with a meta-RR (95% CI) of 0.54 (0.42, 0.69). In subgroup analysis, there were similar protective effects as these results, whether for maternal HBeAg status or for low risk and unclear bias (Table 4).

**Table 4:** **Comparison of immunization effects on extrauterine prevention alone for newborns of HBsAg- and/or HBeAg-positive women** ^&^

**Pregnancy infection status**	**Newborn infection status**	**Detective time**	**Number of included studies**	**Sample size**	**Meta-RR (95% CI)**
Total (HBsAg+ and/or HBeAg+)	HBsAg+	At birth	7	1061	**0.66(0.52,0.84)** ^**3)**^
		7-12 month	12	1451	**0.54(0.42,0.69)** ^**3)**^
		>12 month	7	1214	**0.54(0.42,0.69)** ^**3)**^
	HBsAb+	At birth	4	291	**2.12(1.66,2.70)** ^**3)**^
		7-12 month	8	566	**1.12(1.03,1.22)** ^**3)**^
		>12 month	5	502	1.06(0.96,1.16)^3)^
Subgroup (HBsAg+ and HBeAg+)	HBsAg+	At birth	7	782	**0.75(0.57,0.99)** ^**3)**^
		7-12 month	12	1095	**0.56(0.42,0.75)** ^**3)**^
		>12 month	7	889	**0.55(0.41,0.75)** ^**3)**^
	HBsAb+	At birth	4	291	**3.25(1.35,7.83)** ^**2)**^
		7-12 month	9	443	**1.14(1.05,1.24)** ^**3)**^
		>12 month	5	502	1.06(0.96,1.16)^3)^
Subgroup (HBsAg+ and HBeAg-)	HBsAg+	At birth	1	279	0.59(0.34,1.02)^3)^
		7-12 month	3	356	0.64(0.39,1.06)^3)^
		>12 month	2	325	**0.63(0.41,0.97)** ^**3)**^
Subgroup (low + unclear bias)	HBsAg+	At birth	4	348	0.82(0.58,1.18)^3)^
		7-12 month	10	739	**0.56(0.38,0.82)** ^**3)**^
		>12 month	4	400	**0.43(0.23,0.82)** ^**3)**^
	HBsAb+	At birth	3	221	**4.21(2.70,6.57)** ^**3)**^
		7-12 month	8	566	**1.12(1.03,1.22)** ^**3)**^
		>12 month	4	400	1.05(0.93,1.17)^3)^
Subgroup (high risk bias)	HBsAg+	At birth	3	713	**0.58(0.42,0.81)** ^**3)**^
		7-12 month	2	712	**0.53(0.39,0.72)** ^**3)**^
		>12 month	3	814	**0.56(0.43,0.73)** ^**3)**^
	HBsAb+	At birth	1	70	0.91(0.76,1.09)
		7-12 month	-	-	-
		>12 month	1	102	1.09(0.93,1.27)

^&^(Mother: none/Infants: HBIG + vaccine) vs (Mother: none/Infants: vaccine); ^2)^Random effects model, inverse variance method; ^3)^Fixed effects model, Mantel-Haenszel method; values in boldface indicate statistical significance (*P* < 0.05).

Meta-analysis showed that newborns in the experimental group had a higher amount of protective antibodies at birth and at 7–12 months of age, but not at more than 12 months of age (95% CI including “1”), compared with the control group (Figure 3D). There were 152 newborns in the experimental group and 139 in the control group at birth that were included in four RCTs (Table 4 and Figure 3D). The meta-RR (95% CI) comparing these two groups for newborn anti–HBs-positive rate at birth was 2.12 (1.66, 2.70); a higher level of heterogeneity was observed (*I*^*2*^ = 95%). There were 296 infants in the experimental group and 270 in the control group who had data on their serum anti–HBs-positive status at 7–12 months of age that were included in eight RCTs, with a meta-RR (95% CI) of 1.12 (1.03, 1.22) (*I*^*2*^ = 35%). Five RCTs included data at more than 12 months of age, with a meta-RR (95% CI) of 1.06 (0.96, 1.16). In subgroup analysis, there were similar protective effects as these results between the experimental and control groups for pregnant women with DP that included studies with low risk and unclear bias (Table 4). However, there was lack of data about anti–HBs-positive status when comparing these two groups for newborns of women with SP.

Egger’s test revealed the existence of publication bias when comparing two groups for newborn HBsAg infection rate at more than 12 months of age and for newborn anti–HBs-positive rate at birth (Figure 4C,D). The funnel plots show similar results but with a more skewed shape.

### Other prevention studies

Figure 3E and Figure 3F show a comparison of the effects of immunization between HBV-infected mothers who received HBIG during pregnancy and newborns that received HBIG and HBVac versus newborns that received HBVac. Meta-analysis showed that newborns in the experimental group had a lower infection rate and a higher amount of protective antibodies at each time point than did the control group. Publication bias was not shown because of the low number of included studies.

### Safety analysis

No adverse events—such as fever, rigor, skin rash, inflammation, scleroma at the locally injected area, impairment of renal function, or other discomforts—were found in any of the included studies.

## Discussion

In this study, meta-analysis was used to investigate the clinical effects of different immunization strategies on interrupting MTCT of HBV. The main findings of our study follow.

First, our study found that multiple small doses of intramuscular HBIG injection in HBV-carrying mothers during the third trimester of pregnancy could reduce infants’ HBV infection rate and increase their anti–HBs-positive rate at birth. However, there was no statistical significance in the anti–HBs-positive rate of newborns at more than 7 months of age between the experimental and control groups. Subgroup analysis, such as for DP pregnant women and for the studies without a high risk of bias, showed similar results. The possible mechanism for this is that HBIG administration produces short-term effects, whereas passive HBVac immunization has long-term effects. Furthermore, only one RCT [34] with 3 years’ follow-up supported this view; its meta-RR (95% CI) was 0.33 (0.01, 7.59). Recently, other studies [17,19] have indicated that prenatal HBIG vaccination is effective and improves the immune response for DP pregnant women. Shi et al. [20] and Xu et al. [21] used meta-analyses to show the same viewpoint as these other studies, but Yuan et al. [22] proposed the opposite view. They found that there were no significant differences in newborns between those vaccinated and not vaccinated with HBIG during pregnancy. Our study suggested that multiple small doses of intramuscular HBIG injection during the third trimester of pregnancy still need to be proven by RCTs; this was based on the following considerations. Most of the included studies had an unclear or higher risk of bias and there was clearly publication bias. Extensive HBIG vaccination might lead to immune resistance to HBV strains, which potentially results in the HBVac being ineffective. Additionally, it is possible to produce antigen–antibody immune complexes *in vivo* in pregnant women injected with HBIG that threaten maternal and fetal health.

Second, in pregnant women who carry HBV, passive-active immunization can be efficient for preventing MTCT of HBV [5], especially for DP pregnant women. Notably, most of the RCT studies were carried out before 2000 because immunization strategies were recommended by many countries at that same time. Additionally, many included studies were of low quality, such as a lack of blinding and allocation concealment. Fortunately, subgroup analysis and funnel plots supported the previously mentioned conclusion. There are few studies on SP pregnant women in the study because some quasi-RCT studies without random allocation or with allocation according to willingness were excluded. In SP pregnant women, subgroup analysis showed a lower HBsAg infection rate for newborns who received HBIG and HBVac than those who had just HBVac at more than 12 months of age. This suggests that newborns of SP pregnant women should receive passive-active immunization [81].

Third, more attention should be paid to some neglected issues in the application of clinical trials in vertical MTCT of HBV. Considering the feasibility of trials, many researchers used the method of allocation according to patient willingness [82], not random allocation. Whether pregnant women are willing to be injected with HBIG is dependent on factors such as economic condition, educational level, and HBeAg status. Imbalance of these factors between groups would bias the results. Furthermore, most—except for a few [22,31,54]—of the included studies in our meta-analysis did not describe how to randomly divide study subjects into groups. In addition, blinding and control choice need to be carefully considered; therefore, researchers should increase their cooperation with statisticians and epidemiologists and carefully design clinical trials with them under the guide of the CONSORT criteria before starting a trial.

Our study had the following advantages. (1) Multiple immunization strategies were used to comprehensively investigate, evaluate, and compare strategies. These methods were helpful for minimizing the effect of bias and for improving the accuracy of the study. (2) HBeAg positivity had a close relationship with HBV DNA level. Having access to HBeAg infection status was helpful for controlling bias for evaluating immunization strategy [81]. Wen et al. [83] found that offspring of HBeAg-positive mothers were more likely to be infected and to become chronic carriers than those of HBeAg-negative mothers. This was most likely attributable to the difference in maternal viral load or HBV DNA level. (3) Unlike previous meta-analyses [20,21], the quality of our studies was evaluated using the Cochrane Handbook for Systematic Reviews of Interventions, version 5.1.0, recommended standard. Subgroup analysis of non–high-risk bias facilitated improved appraisal of evidence and led to better health care. In addition, including more studies increased the statistical efficacy.

Our study had the following limitations. (1) Different from extrauterine prevention, intrauterine prevention included these RCTs all from Chinese studies. This limits our results in terms of how to generalize them to other countries. (2) The meta-analysis of SP pregnant women was based on subgroup analysis or non-RCT studies; therefore, these results need to be verified by further RCT studies. (3) Long-term effects in the RCTs were difficult to obtain, especially for certain time points. (4) A lack of sufficient information can bias the results (e.g., mode of delivery, maternal HBV change, laboratory technology, patient HBV DNA level). If umbilical blood is contaminated by maternal body fluids, the error diagnosis of newborn HBV infection could misinform the final results. In addition, it is essential to consider cost-effectiveness analysis, such as the relationship between the cost of multiple small doses of HBIG and protective effects, to evaluate different immunization interventions.

## Conclusions

Although our meta-analysis shows a protective effect of HBIG vaccination of women during the third trimester of pregnancy, this should be further validated by long-term, large-scale randomized trials. In addition, newborns of both DP women and SP women should receive passive-active immunization.

## Additional files

Additional file 1: Table S1. PRISMA 2009 Checklist. Additional file 2: Figure S1. Risk of bias summary for each included study. **A**. Intrauterine prevention. **B**. Extrauterine prevention.
