# Supplementary material for: Immunization interventions to interrupt hepatitis B virus mother-to-child transmission: a meta-analysis of randomized controlled trials
Source: BMC Pediatr. 2014 Dec 20;14:307. doi: 10.1186/s12887-014-0307-2 (PMC4297423; doi:10.1186/s12887-014-0307-2)
Supplement: Additional file 2: Figure S1. — Risk of bias summary for each included study. A. Intrauterine prevention. B. Extrauterine prevention. [file 12887_2014_307_MOESM2_ESM.ppt]

## Slide 1
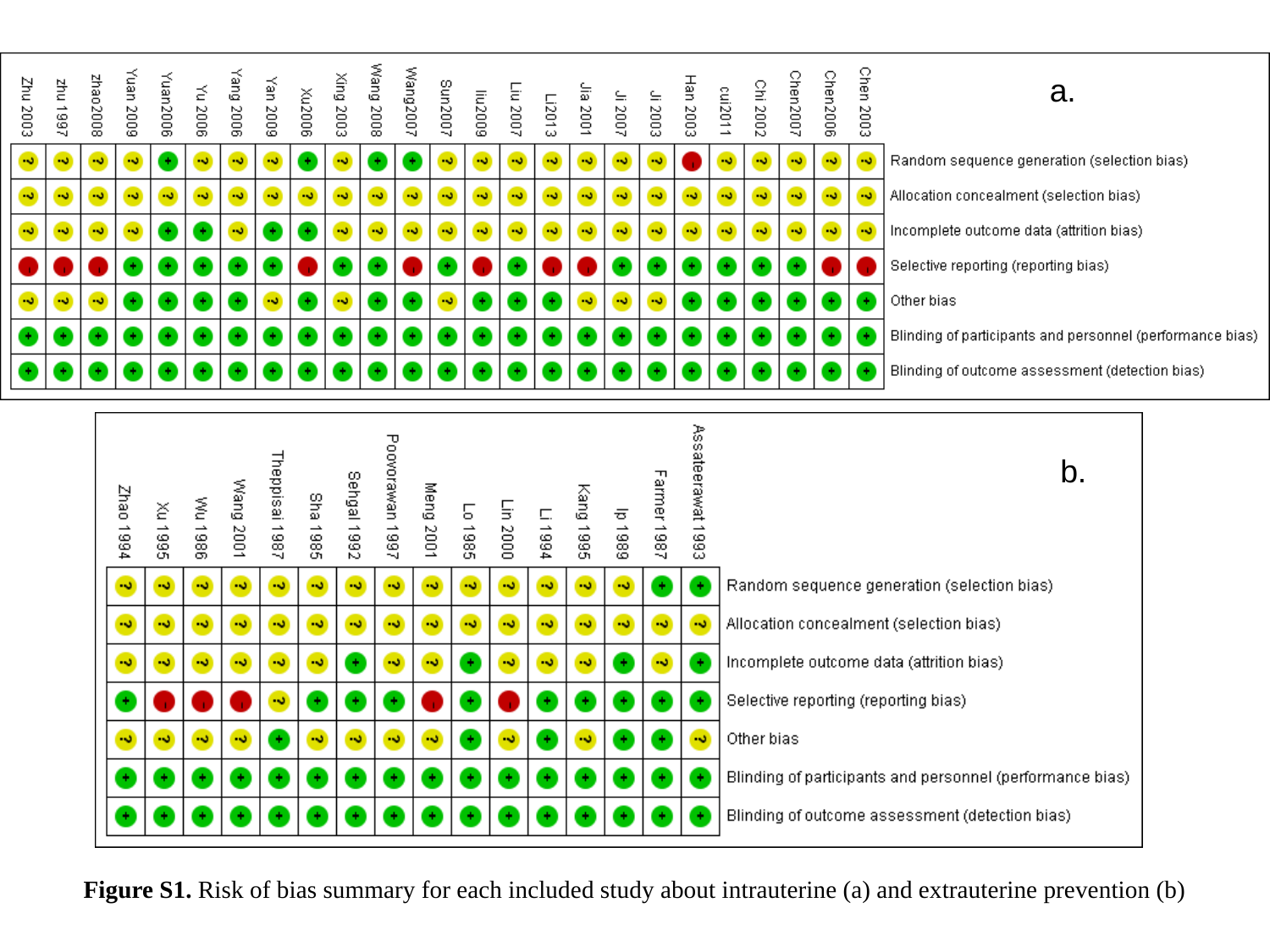

a.
b.
Figure S1. Risk of bias summary for each included study about intrauterine (a) and extrauterine prevention (b)
